# Interventions to reduce dependency in personal activities of daily living in community dwelling adults who use homecare services: a systematic review

**DOI:** 10.1177/0269215514564894

**Published:** 2015-11

**Authors:** Phillip J Whitehead, Esme J Worthington, Ruth H Parry, Marion F Walker, Avril ER Drummond

**Affiliations:** Faculty of Medicine and Health Sciences, University of Nottingham, UK

**Keywords:** Homecare services, personal activities of daily living, re-ablement, restorative homecare, occupational therapy

## Abstract

**Objectives:**

To identify interventions that aim to reduce dependency in activities of daily living (ADL) in homecare service users. To determine: content; effectiveness in improving ability to perform ADL; and whether delivery by qualified occupational therapists influences effectiveness.

**Data sources:**

The Cochrane Central Register of Controlled Trials, MEDLINE, EMBASE, AMED, CINAHL, PsycINFO, OTseeker, PEDro, Web of Science, CIRRIE, and ASSIA.

**Review methods:**

We included: randomised controlled trials, non-randomised controlled trials and controlled before and after studies. Two reviewers independently screened studies for inclusion, assessed risk of bias and extracted data. A narrative synthesis of the findings was conducted.

**Results:**

Thirteen studies were included, totalling 4975 participants. Ten (77%) were judged to have risk of bias. Interventions were categorised as those termed ‘re-ablement’ or ‘restorative homecare’ (*n*=5/13); and those involving separate components which were not described using this terminology (*n*=8/13). *Content* of the intervention and level of health professional input varied within and between studies. Effectiveness on ADL: eight studies included an ADL outcome, five favoured the intervention group, only two with statistical significance, both these were controlled before and after studies judged at high risk of bias. ADL outcome was reported using seven different measures. Occupational therapy: there was insufficient evidence to determine whether involvement of qualified occupational therapists influenced effectiveness.

**Conclusion:**

There is limited evidence that interventions targeted at personal ADL can reduce homecare service users’ dependency with activities, the content of evaluated interventions varies greatly.

## Introduction

People who experience difficulties with personal care tasks such as washing, dressing and feeding may receive support from “homecare services” in which paid care workers attend the person’s home to provide assistance. Traditionally, such care workers have completed these tasks for the person, with homecare provided on a long-term basis.^1,2^ In resource rich countries, demand for homecare has increased^3,4^ to such an extent that it may outstrip supply.^5^ Thus, alternative services have been developed which provide time-limited, intensive input with the specific and explicit aim of enabling people to become independent in personal care activities wherever possible. These alternatives may be called ‘homecare re-ablement’^6^ or ‘restorative homecare’^7,8^ or be provided through other community rehabilitation services.^9^ Such services are believed to enhance independence and quality of life and to lead to reductions in costs^10,11^ but the validity of these various assumptions have not yet been examined via systematic review.

Although these services may share the over-arching aim of reducing dependency in activities of daily living (ADL) there may be considerable differences between what is actually delivered. For example, some re-ablement/restorative homecare services involve paid care workers only^9^ whilst others may involve multidisciplinary input. Therefore it is important to include some description of the content of the intervention evaluated. A distinctive feature of homecare re-ablement is its aim to reduce assistance from paid care workers with personal ADL. Whilst it may appear that people who are discharged without ongoing homecare are better able to manage their own personal care, this may not necessarily be the case (for example, they may be relying on informal caregivers). For this reason, it is important that ability to perform ADL is evaluated as a separate outcome, in addition to the need for ongoing homecare services.

Although occupational therapists have specialist skills in providing interventions targeted at performance in ADL, indeed occupational therapy interventions have been shown to be effective at improving performance in ADL in other contexts,^12–16^ it is currently unclear whether occupational therapy skills are essential to successful homecare re-ablement.^11^ The role and input provided by occupational therapists into these services has been highlighted as a research priority by The Social Care Institute for Excellence.^17^

A previous literature review on restorative approaches to homecare identified only three studies that evaluated interventions embedded within homecare services, and did not describe a formal quality assessment of these studies.^18^ Therefore a systematic review is warranted in order to assimilate the relevant evidence and assess its quality. For the purpose of our review, we were interested in studies that delivered an intervention designed to reduce dependency in ADL for people who were receiving assistance from a paid care worker, compared with provision of routine care where there was no explicit intention to reduce dependency. Our scope was purposely broad in order to identify such interventions that were either: (a) defined using the terms ‘re-ablement’ or ‘restorative homecare’ or (b) provided interventions to homecare users but were not described using these terms.

There were three objectives for our review:

1. To determine what interventions for adult users of homecare services, targeted at reducing dependency in personal ADL, have been provided and evaluated in the literature.
2. To determine the effectiveness of these interventions in relation to individuals’ dependency in ADL.
3. To determine whether interventions involving delivery by occupational therapists differ in their effect to those that do not involve them.

## Method

The review was registered in the PROSEPERO database (CRD CRD42013004163)^19^ and the protocol was published prospectively.^20^ This review conforms to the PRISMA statement.^21^

Randomised controlled trials (RCTs), nonrandomised controlled trials, controlled before and after studies and interrupted time series were all eligible. Participants included individuals, aged 18 years or older, living at home in the community (i.e. not in residential or nursing homes), and in receipt of homecare. For this review, homecare was defined as consisting of one or more weekly visit(s) from a paid care worker to provide assistance with personal ADL. Homecare is distinct from “home healthcare” defined as services offered by qualified professionals including doctors, nurses and allied health professionals to individuals in their own homes. To be included in the review, the homecare service could have involved a healthcare component (e.g. nurse visits) but could not be composed exclusively of healthcare.

Studies were eligible for inclusion if a mixture of assistance with personal (such as washing and dressing) and domestic (such as cleaning) ADL was provided but studies were excluded if all participants received help only with domestic ADL. Studies of participants receiving palliative care were excluded because of the likelihood of physical deterioration and different outcomes.

We included any intervention delivered in or from the participant’s home and designed to reduce dependency in personal ADL and to reduce the need for paid care. We included single component interventions (for example, mono-professional or one-off visits) or multiple components (for example a package provided by a multidisciplinary team). The comparator was defined as a routine homecare service in which assistance with personal ADL was provided but where there was no intention to improve individuals’ performance in this.

The main outcome of interest was performance in personal ADL (including washing, dressing, bathing/showering, feeding, toileting, management of continence, transfers, and basic mobility). Other outcomes included: death; performance in extended ADL (for example, shopping, outdoor mobility); admission to hospital, residential or nursing care homes; falls; mood/morale; health or social care related quality of life; caregiver strain/burden; health economic outcomes; use of health and community services; participant and carer satisfaction with services; and healthcare provider satisfaction with the service. Outcomes were grouped into short term (<6 months), medium term (6 to 12 months) and long term (>12 months).

The following databases were searched for studies published prior to November 2014: the Cochrane Central Register of Controlled Trials, MEDLINE, EMBASE, AMED, CINAHL, Psyc-INFO, Occupational therapy database of systematic reviews and randomised controlled trials (OTseeker), Physiotherapy Evidence Database (PEDro), Web of Science, Center for International Rehabilitation Research Information and Exchange (CIRRIE), and Applied Social Sciences Index and Abstracts (ASSIA). A combination of subject headings and free text terms were used in the search strategy. The search was conducted in English. The search strategy for Medline is shown in appendix one and this was adapted for the other databases.

A three-stage screening process was followed. First, one reviewer (PW) examined titles only and excluded studies evidently not pertinent to the review. Second, the abstracts of all remaining records were screened independently by two reviewers (PW, EW). Thirdly, for remaining records deemed potentially relevant, full-text copies were screened independently and in duplicate by two reviewers (PW, EW). Pre-prepared and piloted forms were used to extract the data and data were independently extracted, in duplicate, by two reviewers (PW, EW). Results were compared and discussed by these two reviewers; any disagreements were resolved in consultation with a third reviewer (AD). Where data were unavailable or unclear, attempts were made to contact authors to obtain and/or verify data.

Two reviewers (PW, EW) independently assessed the methodological quality of the included studies using the criteria developed by the Cochrane Effective Practice and Organisation of Care (EPOC) review group.^22^ Assessment covered sequence generation, allocation concealment, baseline characteristics and measurements, blinding of outcome assessment, completeness of outcome data, selective outcome reporting, and other potential sources of bias. Each of these was rated as being at low, high or uncertain risk of bias. Disagreement between reviewers was resolved by discussion with a third reviewer (AD).

Data were synthesised systematically in a narrative synthesis. Tables and narrative summaries were compiled for characteristics and findings of included studies. Synthesis explored the relationship and findings both within and between the included studies, in line with the guidance from the Centre for Reviews and Dissemination.^23^ Synthesis was conducted by the first author with extensive discussion and final agreement involving all authors.

## Results

**Figure 1.**
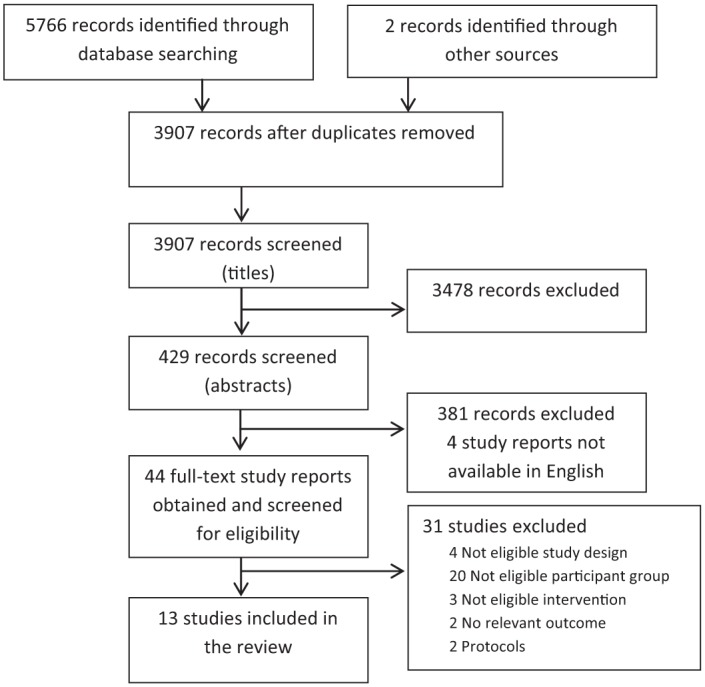
Flow diagram of search process.

The search process is summarised in Figure 1, 13 studies were included.^24–36^ Summary characteristics of the included studies are given in Table 1. Six RCTs and seven controlled before and after studies with a total of 4975 participants were included. Sample size ranged from 74 to 1382, mean 383. The mean size of the RCTs was 276 and 474 for controlled before and after studies.

**Table 1.** Characteristics of included studies.

Country and Study reference	Study type	Participants	Primary outcome	Follow-up months
USA, Feldman^24^	CBA	404 frail elderly and disabled homecare clients.	No primary stated. Home care costs, functioning, depression and satisfaction.	16
England, Glendinning^25^	CBA	1015 adults aged 18 and over receiving homecare or homecare re-ablement.	Health and social care related quality of life.	9 to 12
USA, Gottlieb^26^	CBA	159 adults aged over 60 years, receiving homecare services co-ordinated by a case manager.	No primary. Functional, service and equipment use/satisfaction outcomes.	6
New Zealand, King^27^	RCT (Cluster)	186 adults aged 65 years and over, receiving assistance from homecare agency.	Health Related Quality of Life (SF-36).	7
Australia, Lewin^28^	CBA	200 adults aged 60 and over referred for homecare assistance with domestic or personal care tasks.	No primary stated. Functional and service outcomes.	12
Australia, Lewin^29^	RCT	750* adults aged 65 and over, referred for homecare assistance with personal care.	Service outcome - use of on-going homecare (for personal care).	12
USA, Marek^30^	CBA	85 clients receiving state funded homecare program.	No primary stated. Cognition, ADL, depression, pain, dyspnea, medication management.	12
Canada, Markle-Reid^31^	RCT	126 adults aged 75 and over, eligible for ‘personal support’ services.	Functional Status and Quality of Life (SF-36).	6
Canada, Markle-Reid^32^	RCT	288 adults aged 75 and over, eligible for ‘personal support services’.	Functional Status and Quality of Life (SF-36).	6
Canada, Markle-Reid^33^	RCT	101 community-living stroke survivors using homecare services.	Health Related Quality of Life and functioning (SF-36).	12
New Zealand, Parsons^34^	RCT (Cluster)	205 adults aged 65 and over, newly referred for homecare.	Health Related Quality of Life (SF-36).	6
USA, Tinetti^35^	CBA	1382 adults aged 65 years and over, in receipt of Medicare-covered homecare.	Remaining at home, functional status, duration and intensity of home care episode.	1 month (approx.)
Sweden, Zingmark^36^	CBA	74 adults aged over 65years in the process of applying for help with bathing.	Ability to perform ADL (ADL Taxonomy).	4 (15 weeks)

RCT: randomised controlled trial; CB: controlled before and after study.

* 750 included for service outcomes, 300 included for user outcomes (i.e. ADL ability).

Findings on risk of bias are shown in Table 2. Ten of the 13 studies (77%) were judged to be at high risk of bias in at least one domain, i.e. there was a high risk in the majority of the studies in the review. Only one study was judged to be at low risk of bias in all domains. Selection bias was the most common type of bias. The seven controlled before and after studies were all rated at high risk of selection bias due to the allocation sequence generation and concealment of allocation procedures, in accordance with the criteria used.^22^ This was due to the non-random methods used to allocate participants to treatment or control groups.

**Table 2.** Risk of bias summary.

Study	a	b	c	d	e	f	g	h	i
Feldman^24^	H*	H*	U	H	U	U	U	L	L
Glendinning^25^	H*	H*	H	L	H	U	L	L	L
Gottlieb^26^	H*	H*	H	H	U	H	U	U	L
King^27^	L	L	L	L	L	L	U	L	L
Lewin^28^	H*	H*	L	H	L	H	L	L	L
Lewin^29^	L	H	L	H	L	H	L	H	U
Marek^30^	H*	H*	H	H	H	U	L	L	L
Markle-Reid^31^	L	L	L	L	L	U	L	L	L
Markle-Reid^32^	L	L	H	L	L	L	L	L	L
Markle-Reid^33^	L	L	L	L	L	L	L	L	L
Parsons^34^	L	U	L	H	L	L	L	H	L
Tinetti^35^	H*	H*	H	L	L	H	L	L	L
Zingmark^36^	H*	H*	H	L	U	H	L	L	L

a: Selection bias (sequence generation); b: selection bias (sequence concealment); c: selection bias (baseline measurements); d: selection bias (baseline characteristics); e: attrition bias (outcome data); f: detection bias (blinded assessor); g: performance bias (contamination of intervention/control); h: reporting bias (reporting of all outcomes) i: other bias.

H: high risk; l: low risk; u: unclear risk.

* Automatically rated high risk due to study type.

The first objective was to describe the interventions that have been provided and evaluated. For the purpose of analysis, studies were divided between those that were termed ‘re-ablement’/‘restorative homecare’ (*n*=5) and those in which community based interventions targeting reduced dependence in personal ADL were provided, but not described using this terminology (*n*=8). Table 3 provides a summary overview of each intervention.

**Table 3.** Summary of interventions.

Intervention	Study	Details
Re-ablement/ Restorative homecare	Glendinning^25^, King^27^, Lewin^28,29^, Tinetti^35^	A programme or package of homecare where there was a ‘re-ablement/restorative’ philosophy in which the aim was to improve ability to manage activities independently. This involved a series of different practices and the exact combination of which varied within and between studies and sites. Services were usually time limited, goal-focussed and involved a different approach by paid care workers.
Nurse-led health promotion/care coordination	Marek^30^, Markle-Reid^31,32^	A registered nurse acted as a named contact for the participant, coordinated services and implemented strategies to bolster health and wellbeing such as: providing education and monitoring illnesses.
Cluster care	Fedlman^24^	Reorganisation of homecare services into teams to deliver care to ‘clusters’ of individuals in a locality rather than one-to-one blocks of time. Reduced contact time meant that care workers’ role was based on specific tasks rather than time allocated.
Assistive technology	Gottlieb^26^	Assessment by a case manager followed by the provision of assistive devices (equipment) to increase independence with ADL (meals, bathing, toileting, dressing, mobility). Case managers received training from occupational therapists.
Specialist inter-professional stroke care	Markle-Reid^33^	Comprehensive rehabilitation services provided by multidisciplinary team with specialist stroke training and expertise involving: rehabilitation, education, support and case management.
Goal-setting	Parsons^34^	Use of a goal-facilitation tool to set objectives for the homecare episode was implemented by a trained assessor and then goals were passed onto the homecare agency staff.
Occupational therapy bathing intervention	Zingmark^36^	Assessment of individual needs by an occupational therapist. Interventions were then tailored in order to maximise their performance in bathing.

The five studies that termed the intervention ‘re-ablement’ or ‘restorative homecare’ interventions^25,27–29,35^ shared certain features in that they: involved a team approach; incorporated multiple components within the intervention including: goal-setting; support, education and advice; assistance with, training or practice of activities; and sometimes falls prevention work. Some also included training of the care workers in relevant skills. The specific content and mix of components of varied between studies and appeared to be adapted for individual participants within studies. Three of these studies involved face-to-face contact from therapists, nurses and paid care workers;^28,29,35^ one involved paid care workers with nurses acting in a coordinator role;^27^ and one was mixed with some sites in the study having no health professional input at all within the team.^25^ The majority of studies involved input from one or more health professionals sometimes providing training to the paid care workers.

The remaining eight studies evaluated the interventions targeting ADL independence not labelled as re-ablement or restorative homecare and mostly involved specific single components: nurse-led health promotion or care coordination (*n*=3);^30–32^ cluster care (*n*=1);^24^ assistive technology (*n*=1);^26^ goal-setting by a trained assessor using a tool (*n*=1);^34^ and occupational therapy bathing intervention (*n*=1).^36^ One involved input from a specialist multidisciplinary team: inter-professional stroke care (*n*=1).^33^ The interventions evaluated in these eight studies overlap with one another, and with the interventions provided within re-ablement or restorative care services. For example, the occupational therapy bathing intervention^36^ involved provision of assistive technology which was delivered as a standalone component in another study^26^ and as part of several of the re-ablement/restorative care interventions.

The thirteen included studies varied widely in the duration of the intervention, from single-contact assessments,^26^ to service level changes which were not time limited.^24,33^ Interventions labelled re-ablement or restorative care tended to involve shorter durations ranging from just under four weeks^35^ up to 12 weeks;^28,29^ although one was not time limited.^27^ The intensity (i.e. how much contact each service user received) varied both within and between studies in all categories. However, little information was given as to how intensity was decided for each individual user.

Key components across the 13 studies were:

- Goal-setting at the beginning of the homecare episode;
- Repetitive practice and/or grading of activities;
- Co-ordination or case management of the homecare episode by an individual or team
- provision of equipment (assistive devices);
- Re-organisation of services to maximum efficiency based on approach, tasks, time or specialist knowledge.

Key differences between the interventions related to:

- The number and combination of components;
- The combination of people delivering the intervention (i.e. single discipline or multidisciplinary team; professionally qualified or non-qualified);
- The duration and intensity of the intervention.

(These components have been synthesised across all studies and were not present in each individual study).

The second objective was to determine effectiveness of the interventions in improving indi-viduals’ performance in ADL. Table 4 summarises this outcome for each study. Only eight studies^24–26,28,30,35,36^ reported this outcome using an ADL measure; seven different measurement methods were used. Some of these were not standardised: some studies used actual reports of ability or completion of tasks, others were based on users’ perceived difficulty in completing tasks. Of these eight, two showed statistically significant improvement in ADL ability in favour of the intervention group (indicated by * in the far right column of Table 4), one a non-significant improvement in the intervention group compared to the control group, and one showed no difference between the groups, although the authors noted possible contamination of the control group.^29^ A further two reported the non-significant change scores only. It was therefore impossible to extract group or change data. The remaining two reported ADL measurement for individual activities (such as ‘dressing’) separately. This made it impossible to determine an overall effect for these studies,^25,36^ although more activities improved in the intervention group compared to the control group. Overall, findings on ADL favoured the intervention group in five of the eight studies.

**Table 4.** Summary of effect on ADL performance/physical function (by study).

Study reference	Measure used	Time point(s)	Effect	Sig
Feldman^24^	Participant reports of difficulty.	LT	Change scores only reported.	
			No difference between groups.	
Glendinning^25^	List of ADL activities .	MT	No overall scale score.	
			Higher percentage in intervention group gained the ability to: walk outside, bath or shower, dress and undress.	
Gottlieb^26^	Client’s perceived difficulty in ADL.	MT	Change scores only – bathing and dressing.	
			No differences between groups.	
Lewin^28^	ADL scale based on Modified Barthel Index.	ST	Significant difference in mean change score (favours intervention) at 3 months z= -3.71, *P*< 0.001 and 12 months z= -2.90, *P* = 0.004, adjusted for baseline differences.	*
		MT		
Lewin^29^	ADL scale based on Modified Barthel Index.	ST	No significant difference between the intervention group (M= 11.87) and control group (M=12.65) at 3 months.	
		MT	No significant differences between the intervention group (M= 12.11) and control group (M= 12.82) at 12 months.	
			Data were obtained from authors – SD not given.	
Marek^30^	Five ADL items from minimum dataset for homecare used.	MT	No significant difference between the intervention group (M=1.8; SD= 4.3) and the control group (M= 0.4; SD= 1.3); *P*= 0.65, at 6 months.	*
			Significant difference (favours intervention) between the intervention group (M= 2.1; SD= 4.7) and the control group (M= 3.3; SD= 4.7); *P*= 0.01, at 12 months.	
Tinetti^35^	Self-care ADL score.	ST	Mean self-care score better (not significant) in intervention group (adjusted for baseline difference) t=-1.81, *P*= 0.07.	
Zingmark^36^	ADL taxonomy.	ST	Of 19 ADL activities, seven showed significant improved in both groups and six activities in the intervention group only (walking inside, walking in neighbourhood, getting clothes from wardrobe, washing hair, combing hair, and manicuring).	
King^27^	SF-36 physical component	MT	Change from baseline to 7 months favours intervention (not significant)	
			2.6 CI -1.5, 6.6 P= 0.22.	
Markle-Reid^31^	SF-36 physical function	MT	Significant difference between the intervention group (M= 39.20; SD= 27.40) and the control group (M= 26.30; SD= 22.80); t=2.480, *P*= 0.015.	*
Markle-Reid^32^	SF-36 physical function	MT	Difference in mean change score favours intervention (not significant) -5.39	
			CI -11.13, 0.35, *P*=0.065.	
Markle-Reid^33^	SF-36 physical function		Difference in mean change score favoured intervention (not statistically significant but authors argued that this was clinically significant) 5.87 CI -3.98, 17.73, *P*=0.24.	
Parsons^34^	SF-36 physical component.	MT	Significant difference in inter-group change from baseline (I: 44.45 (3.52) to 54.04 (3.52) C: 52.08 (3.42) to 51.31 (3.42) P=0.0002). Linear mixed methods model used.	*

SF-36: Short Form 36; ST: short-term, <6 months; MT: medium-term 6 to 12 months; LT: long-term > 12 months.

* Significant.

The remaining five studies^27,31–34^ did not report a specific ADL measure, instead reporting the Short Form-36 (SF-36) which includes a physical functioning component. Physical functioning ability may be an important outcome for this population group and may provide a broad indication as to their ability to perform ADL, although this should not be regarded as a substitute for an ADL outcome. All these five showed an effect in favour of the intervention group, which was statistically significant in two studies. It was considered important to include these studies as the population were in receipt of homecare for assistance with personal activities of daily living. If we had excluded them we would have omitted important studies from our analysis.

Findings on the additional outcomes are available in Appendix 2 (online supplementary file). Eleven reported number of deaths; eight reported quality of life; seven reported admissions to hospital, nursing or residential homes; seven reported the amount of paid carer input; six reported ability to perform extended activities of daily living; six reported participant mood/morale; five reported health economic outcomes; and one reported on participant satisfaction. None of the studies included data on falls, carer strain, or provider satisfaction with services.

Seven studies reported on paid care worker input after intervention. All found a significant difference between the intervention and control groups in terms of those requiring care, those requiring a reduction or discontinuation of care, or the costs of ongoing care.^24–29,36^ That is, service users were being provided with less care or less costly care at the final follow-up point. This is an important finding, and suggests that these interventions can reduce the amount of ongoing homecare required. Whether or not this is associated with improvement in ADL independence is not known.

Five studies reported a significant effect in favour of the intervention group, in health related quality of life.^25,27,31,32,34^ One showed no effect,^33^ and two did not provide overall scores.^29,36^ Thus, overall there is some evidence that these interventions can improve health related quality of life, but there are discrepancies between studies in the way the data has been analysed and reported. Two studies showed a significant effect in favour of the intervention group in ability to perform extended activities of daily living;^28,35^ four studies showed no significant difference.^24,26,27,29^

The third objective was to determine if interventions involving occupational therapists differed from those not involving occupational therapists. Occupational therapists were involved in the interventions in seven studies.^25,26,28,29,33,35,36^ Only one of these consisted solely of input provided by a qualified occupational therapist.^36^ In the others occupational therapists provided training to those who delivered the intervention,^26^ or delivered interventions as part of a multidisciplinary team alongside nurses, home care workers, and physiotherapists.^28,29,33,35^ Where occupational therapists formed part of a multidisciplinary team, the exact detail of their involvement in delivering these interventions was unclear and they appear not to be involved with every participant. It was therefore impossible to determine whether interventions involving occupational therapists led to different outcomes to those that did not.

## Discussion

Overall, there is some evidence that interventions aiming to improve ability to independently perform ADL are effective for a population of homecare service users, in comparison to standard homecare services in which assistance is provided with personal care tasks. However, although there is evidence that these interventions may improve this outcome, there is widespread variation in the type and content of the intervention and the method of evaluation used. There is also a risk of methodological bias within the majority of the included studies. Notwithstanding this, the majority of studies showed an effect in favour of the intervention group and this was statistically significant in two studies. There is also evidence that these interventions can reduce the use of, and costs associated with, ongoing care services, particularly homecare. This finding is consistent with the wider literature.^10,18^ There is also some evidence that these interventions improve health related quality of life.

An important finding is that different measurement scales were used for the outcome of performance in personal ADL. This is a key consideration for further research in this area. Eligibility for homecare services is primarily based upon the need for assistance from a paid care worker, usually for personal care. Thus, ability to perform these tasks is an important outcome of intervention. However, several studies focused on health related quality of life, using the EQ5D^25^ or Short- Form 36 (SF-36)^27, 31–34^ with no specific ADL outcome measure. We suggest that specific standardised personal ADL measures should be used at baseline and at all outcome time points in order to capture the effect of the intervention. As Ferrucci et al.^37^ note, “improvements in functional status may not translate into well-being and quality of life” (pp. 627).

A further objective of this review was to determine whether interventions involving occupational therapists led to improved outcomes for users when compared to interventions without this professional input. Occupational therapists were involved in a number of interventions, primarily as members of a multidisciplinary team. Only one study specifically compared a standalone occupational therapy bathing intervention with a control group.^36^ Participants in that study showed improved ability to carry out certain activities of daily living, and a significant reduction in their use of ongoing homecare services. However, this was not a randomised trial and therefore further research is warranted.

A purposely broad scope was adopted for this review and this is both a strength and a limitation. It is a strength insofar as it allowed us to provide an overview of those interventions which might be considered under the umbrella of ‘re-ablement’. This overview will be relevant to practitioners working in re-ablement or restorative homecare who may wish to review the evidence for individual components of their practice with this population group. However, the weakness of our broad perspective was that we included studies with clinical and methodological heterogeneity and therefore a meta-analysis was not possible. Whilst a narrower focus would have identified a smaller number of studies these may also have been unsuitable to combine due to the methodological differences and biases identified. Thus, the broad scope of this review facilitated the identification of enough studies to address the three overarching review objectives.

Although we believe that the extensive search strategy was comprehensive and identified all relevant studies, it is possible that some studies were not identified during the search process. In particular, non-randomised studies are known to be difficult to identify because search terms are not well defined for electronic databases, thus there can be less confidence that all relevant studies of this type have been located. Additionally, all the searches were conducted in English and so studies without an English abstract were not identified. Four papers were also excluded at the full-text screening phase as they had an English abstract but no English translations were available.

An additional limitation was the difficulty in defining the homecare user population. In this review the definition “adults living in the community and receiving assistance from a paid care worker for personal care” was used. However, there are widespread variations between countries in the service structures and organisation of homecare services^38^ and it was sometimes difficult to determine the exact characteristics of the homecare service user population when screening the studies for inclusion at each stage of the review. Furthermore, in some studies, participants may have received a mixture of assistance with personal and domestic ADL, and it was difficult to determine whether they all received assistance with personal care.

The impact of these difficulties was minimised by adopting an overly inclusive strategy between each review stage, and by having two reviewers to screen the abstracts and full-text study reports. However, it is possible that studies may have been missed, particularly if the content or structure of the homecare service was not well described in the paper. It is also possible that some of the homecare services did not actually provide paid care worker assistance to all participants and included those who were eligible to have this assistance (particularly the studies from North America). Furthermore, there may be widespread differences within services themselves in terms of how the re-ablement’ intervention is delivered for individual participants. Thus, it is especially important that the content of re-ablement and restorative homecare interventions are adequately described in the literature in order to facilitate comparisons between services.

This is the only review to date that has collated these studies systematically and therefore has provided the only synthesis of this type. Furthermore, this is the first review to analyse the outcome of ability to perform ADL, which is an important outcome for the homecare user population. Addi-tionally, our findings are consistent with a previous review carried out by Ryburn et al.^18^ and with wider summaries in the literature on re-ablement.^10^ In particular, findings about the reduction in ongoing care services and improvements in health related quality of life are similar to previous research. Whilst this review suggests that interventions can reduce dependency in ADLfor this population group, further research is needed. We believe that it is essential to directly and separately measure ADL ability as well as quality of life and use of ongoing homecare services, in this population. Further research should explore which specific components of re-ablement are most effective, including the role of occupational therapy and other health professional input.

**Clinical messages**

- Homecare services incorporating interventions targeted at personal ADL can improve an individual’s ability to carry out these activities independently.
- Re-ablement or restorative homecare interventions commonly involve more than one component. Content of interventions varies widely. The optimum configuration is currently unclear.

## Supplementary Material

Supplementary material
