# Supplementary material for: Interventions to reduce dependency in personal activities of daily living in community dwelling adults who use homecare services: a systematic review
Source: Clin Rehabil. 2015 Nov;29(11):1064–76. doi: 10.1177/0269215514564894 (PMC4607918; doi:10.1177/0269215514564894)
Supplement: Supplementary material [file CRE564894.pdf]

Appendix 1: Search Strategy

1. home care services/
2. home health aides/
3. homemaker services/
4. homecare.tw.
5. (home$ adj1 (care$ or treat$ or help$)).tw.
6. (home$ adj2 (service$ or aid$)).tw.
7. domiciliary.tw.
8. 1 or 2 or 3 or 4 or 5 or 6 or 7
9. activities of daily living/
10. self care/
11. independent living/
12. (function$ adj1 (independ$ or abilit$)).tw.
13. (self adj1 care).tw.
14. (activit$ adj2 daily).tw.
15. (restorat$ adj2 (care$ or model$ or service$ or home$)).tw.
16. (re-able$ or reable$ or re-enablem$ or enablem$).tw.
17. goals/
18. (goal$ adj2 (set$ or treat$ or therap$)).tw.
19. 9 or 10 or 11 or 12 or 13 or 14 or 15 or 16 or 17 or 18
20. 8 and 19
21. randomized controlled trial.pt.
22. controlled clinical trial.pt.
23. (control$ adj2 trial).tw.
24. intervention studies/
25. experiment$.tw.
26. (time adj1 series).tw.
27. (pre test or pretest or posttest or post test).tw.
28. random allocation/
29. intervention?.tw.
30. evaluation studies/
31. comparative study.pt
32. 21 or 22 or 23 or 24 or 25 or 26 or 27 or 28 or 29 or 30 or 31
33. 21 and 32
34. nursing home/
35. 33 not 34
36. limit to adults

Appendix 2: Systematic Review Secondary Outcomes

Study	Death	EADL	Admissions	Falls	Mood	HrQoL SCrQoL
Feldman[24]	I: 15% (of 229)<br>C: 14% (of 175)	Change scores only – no difference between groups	Not Reported	Not Reported	CES-D<br>No data – but reported no differences between groups	Not Reported
Glendinning[25]	I: 11% (74/654)<br>C: 12% (44/361)	Not Reported	Not Reported	Not Reported	Not Reported	Significant improvement in EQ5D score in re-ablement group (P<0.001). No change in control group.<br><br>No significant differences in ASCOT scores.
Gottlieb[26]	Not Reported	Change scores – no differences between groups	Not Reported	Not Reported	Not Reported	Not Reported
King[27]	**4M**<br>I: 2% (2/93)<br>C: 0% (0/93)<br><br>**7M**<br>I: 3% (3/93)<br>C: 2% (2/93)	**NEADL - 4M**<br><br>I: 44.3 (0.9)<br>C: 45.0 (1.0)<br><br>**NEADL– 7M**<br><br>I: 44.2 (1.0)<br>C: 44.4 (1.1)	**Residential**<br><br>**4M**<br>I: 3% (3/93)<br>C: 2% (2/93)<br><br>**7M**<br>I: 4% (4/93)<br>C: 2% (2/93)	Not Reported	Not Reported	SF-36 (overall) Between arm difference in change 3.8 (0.0, 7.7) P= 0.05.
Lewin[28]	**3M**<br>I: 4% (4/100)<br>C: 4% (4/100)<br><br>**12M**<br>I: 11% (11/100)<br>C: 11% (11/100)	***PAF – IADL***<br><br>**3M**<br>I: 14.8(3.7)<br>C: 14.9(4.1)<br><br>**12M**<br>I: 14.0 (2.8)<br>C: 14.5 (3.9)	**Residential**<br><br>3M<br>I: 1%(1/100)<br>C: 2%(2/100)<br><br>12M<br>I: 2% (2/100)<br>C: 4% (4/100)	Not Reported	PGMS<br><br>3M<br>I: 10.4 (3.6)<br>C: 11.0 (3.7)<br><br>12M<br>I: 10.8 (3.4)<br>C: 10.9 (3.6)<br><br>Significant difference in change (2.04, P=0.041)	Not Reported
Lewin[29]	**3M**<br>I: 5% (17/375)<br>C: 7% (25/375)<br><br>**6M**<br>I: 17% (65/375)<br>C: 19% (72/375)	Obtained from authors<br><br>No difference between groups	**Hospital / Residential / Hospice**<br><br>3M<br>I: 13% (49/375)<br>C: 7% (27/375)<br><br>6M<br>I:13% (49/375)<br>C: 14% (52/375)	Not Reported	Not Reported	Collected but not presented in paper. No differences reported.

Study	Death	EADL	Admissions	Falls	Mood	HrQoL SCrQoL
Marek[30]	I: 11% (6/55)<br>C: 7% (2/30)	Not Reported	**Nursing Home**<br><br>I: 4% (2/55)<br>C: 23% (7/30)	Not Reported	Mood indicators – Minimum dataset<br><br>I: 0.3 (0.6)<br>C: 0.7 (1.5)<br><br>No difference	Not Reported
Markle-Reid[31]	I: 10% (6/62)<br>C: 11% (7/64)	Not Reported	**Hospital Admission**<br><br>I: 16%(of 62)<br>C: 24% (of 64)	Not Reported	CES-D<br><br>I: 8.61 (6.98)<br>C: 13.64 (12.04)<br>P=0.02	SF-36<br><br>Split into component scales – significant improvement in summary scales in intervention group.
Markle-Reid[32]	Group data not reported.	Not Reported	Not Reported	Not Reported	CES-D<br>Difference in mean change in favour of I: 2.72 (0.39, 5.07), P 0.022	SF-36<br><br>Split into component scales
Markle-Reid[33]	I: 8% (4/52)<br>C: 6% (3/49)	Not Reported	Not Reported	Not Reported	CES-D<br><br>I: 11.40 (11.95)<br>C: 12.62 (10.9)<br><br>No difference	SF-36<br><br>Split into component scales
Parsons[34]	I: 0% (0/108)<br>C: 4% (4/97)	Not Reported	Not Reported	Not Reported	Not Reported	SF-36 (overall) Intergroup difference in change from baseline P <0.0001 in favour of Intervention.
Tinetti[35]	I: 0.4% (3/691)<br>C: 0.1% (1/691)	**Home Mgmt**<br><br>I: 9.5 (2.9)<br>C: 9.2 (3.0)	**No longer at home**<br><br>I: 18% (122/691)<br>C: 29% (201/691)	Not reported	Not reported	Not reported
Zingmark[36]	I: 9% (5/55)<br>C: 9% (3/32)	Not Reported	Nursing Home<br>I: 2% (1/55)<br>C: 0% (0/32)	Not Reported	Not Reported	EQ5D Graphically displayed (no data)
**Total (/13)**	**11**	**6**	**7**	**0**	**6**	**8**

Study	Health Economic	Caregiver Strain	On-going Homecare	Participant Satisfaction	Provider Satisfaction
Feldman[24]	$2,900 annual cost saving per person in intervention group	Not Reported	Average of 6 less hours per week in intervention group.	Participants at intervention site significantly less satisfied with service.	Not Reported
Glendinning[25]	Re-ablement associated with significant decrease in subsequent social care costs.<br><br>High probability of cost effectiveness.	Not Reported	Cost data, not hours or per participant.<br><br>Intervention participants used less social care services during follow-up period.	Not Reported	Not Reported
Gottlieb[26]	Not Reported	Not Reported	Significantly greater increase in use of formal service in control group (change scores only – no data).	Nor Reported	Not Reported
King[27]	Not Reported	Not Reported	Reduced Support<br><br>I: 29% (22/76)<br>C: 0% (0/81)	Not Reported	Not Reported
Lewin[28]	Not Reported	Not Reported	Reduced/ Discontinued<br><br>3M<br>I:66% (66/100)<br>C: 11% (11/100)<br><br>12M<br>I: 65% (65/100)<br>C: 26% (26/100)	Not Reported	Not Reported
Lewin[29]	Not Reported	Not Reported	Needing on-going 'personal care'<br><br>3M<br>I: 28% (103/375)<br>C: 64% (238/375)<br><br>6M<br>I: 18% (67/375)<br>C: 40% (151/375)	Not Reported	Not Reported
Marek[30]	Not Reported	Not Reported	Not Reported	Not Reported	Not Reported
Markle-Reid[31]	Higher costs related to intervention; lower acute hospitalisation costs.	Not Reported	Not Reported	Not Reported	Not Reported

Study	Health Economic	Caregiver Strain	On-going Homecare	Participant Satisfaction	Provider Satisfaction
Markle-Reid[32]	No differences in health and social services cost at 6months.	Not Reported	Not Reported	Not Reported	Not Reported
Markle-Reid[33]	Higher total per-person mean cost of health services in the intervention group.	Not Reported	Not Reported	Not Reported	Not Reported
Parsons[34]	Not Reported	Not Reported	Not Reported	Not Reported	Not Reported
Tinetti[35]	Not Reported	Not Reported	Not Reported	Not Reported	Not Reported
Zingmark[36]	Not Reported	Not Reported	I: 30% (14/46)<br>C: 75% (21/28)	Not Reported	Not Reported
**Total (/ 13)**	**5**	**0**	**6**	**1**	**0**

Key:
M = Months
I = Intervention
C = Control
Deaths and admissions data is cumulative where there is more than one time point.
Differences in data presentation reflects the different ways in which the data was reported (and what could be extracted) from the original papers.
